# Thymic Carcinoma: A Review

**DOI:** 10.3389/fonc.2022.808019

**Published:** 2022-04-08

**Authors:** Doaa Alqaidy, Cesar A. Moran

**Affiliations:** ^1^Department of Pathology and Laboratory Medicine, University of California San Francisco, San Francisco, CA, United States; ^2^Department of Pathology, MD Anderson Cancer Center, University of Texas, Houston, TX, United States

**Keywords:** thymus, carcinoma, mediastinum, thymoma, staging

## Abstract

The diagnosis of thymic carcinoma may pose significant problems not necessarily in the histopathological diagnosis but rather in assigning the thymus as specific origin. Often the tissue available for interpretation is obtained *via* a mediastinocopic biopsy, which raises two different issues -minimal tissue and lack of specific features to make a carcinoma of thymic origin. In addition, if to that conundrum we add that there is no magic immunohistochemical stain that will unequivocally lead to the interpretation of thymic carcinoma, then we are left with a true clinical-radiological-pathological correlation. In this review, we will highlight some of those challenges that diagnostic surgical pathologists may encounter in the histopathological assessment of thymic carcinoma as well as in the staging of these tumors.

## Introduction

Primary thymic carcinoma in general practice represents a small percentage of thymic epithelial neoplasms (1). Contrary to thymoma, which is much more common, and a tumor in which the histopathological features, in the majority of cases, allow for the tumor to be not only diagnosed easier but also be placed in specific anatomical area, the diagnostic features of thymic carcinoma are not specific, and its diagnosis requires complete clinical and radiological correlations.

Historically, the entity that we currently recognize as thymic carcinoma had not been acknowledged in the literature until Shimosato et al. (2) reported a series of cases in which the authors provided enough evidence to define the tumor as of arising from the mediastinal compartment. Followed that series of cases, there have been numerous other reports; however, the only two series that have reported more than 60 cases of primary thymic carcinoma are the ones presented by Suster-Rosai (3) and Moran et al. (4), which together will gather only 125 patients with thymic carcinoma and if to that we may add that at least in the Suster-Rosai series, the authors also included some neuroendocrine carcinomas, then we have the two largest series with even fewer non-neuroendocrine thymic carcinomas. Nevertheless, other series consisting of fewer cases have provide important clinical and pathological information, which over the years has expanded not only the clinical knowledge of thymic carcinoma but also has expanded the histopathological spectrum of these tumors (5).

## Clinical Features

Similar to what occurs with the histopathological features of thymic carcinoma —non-specific, the clinical features of patients with these tumors are also rather non-specific. The tumor has been described in a wide range of individuals ranging from very young to older patients, reports of thymic carcinoma in association with some autoimmune diseases or paraneoplastic syndromes including myasthenia gravis have been presented in the literature. However, we consider that those reports are likely coincidental occurrences rather than true link associations. Thymic carcinoma does not appear to have a strong association with Myasthenia Gravis or other autoimmune disease that have been reported in association with thymomas (3, 4, 6). More recently NUT carcinoma, which is another tumor that may occur in the midline appears to affect rather younger individuals. However, the morphology of the tumor is that of a poorly differentiated carcinoma, which requires FISH analysis to properly designate the tumor as NUT carcinoma (7).

The ideal clinical and radiological features of patients with thymic carcinoma should be that of a patient without any prior history of carcinoma and presenting with an anterior mediastinal mass.

In terms of survival, if we compare the two largest series of thymic carcinomas, we can see some differences. In the Suster-Rosai experience (3), the 5-years survival was recorded at 33%, while in the Weissferdt-Moran (4), it was recorded at 65%. It is possible that in the Suster-Rosai series of cases the survival may have been influenced by including neuroendocrine neoplasms, while in the Weissferdt-Moran, those tumors were excluded. Nevertheless, survival will be influenced not only by the histology of the tumor but also by the staging at the time of diagnosis.

## Pathological Features

The gross features of thymic carcinoma only rarely will provide clues of the possibility of thymic carcinoma, as the tumor may show areas of necrosis, hemorrhage, and infiltrative borders. However, those parameters are not specific for thymic carcinoma and may be seen in some cases of thymoma (8). Similarly, the tumor may also show cystic changes but once again, cystic changes are much more common in thymoma than thymic carcinoma. The gold standard to arrive at specific diagnosis is on histopathological evaluation.

**Figure 1:**
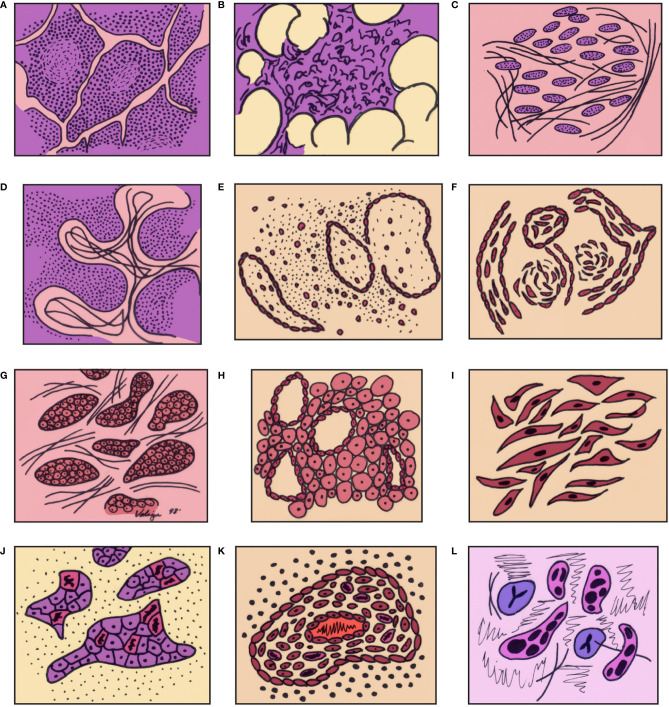
Graphic illustration of the spectrum of differentiation of thymic epithelial neoplasms from normal thymus through high grade thymic carcinoma: **(A)** thymus in a child, **(B)** thymus in the adult, **(C)** thymus with involutional changes, **(D)** thymoma (lymphocyte rich – WHO type B1), **(E)** Thymoma (mixed cellularity – WHO type B2), **(F)** Spindle cell thymoma (WHO type A), **(G)** atypical thymoma – preservation of organotypical features, **(H)** atypical thymoma (perivascular spaces), **(I)** Atypical spindle cell thymoma, **(J)** thymic carcinoma – loss of organotypical features, **(K)** thymic carcinoma – inflammatory reaction, **(L)** thymic carcinoma – cellular atypia and mitotic activity in epithelial cells. (with permission from Dr. Moran copyright ^©^ Dr. Moran).

The histopathological features of thymic carcinoma can be illustrated in a rather diagrammatic way taking the normal thymus as a starting point and following it down to the changes that may be seen in thymoma and further into thymic carcinoma (**Figure 1**). We consider that all those changes are part of the spectrum that thymic epithelial neoplasms. From the light microscopic point of view, the features of thymic carcinoma can be summarized based on the cellularity of the tumor or based on the growth pattern; thus, tumors can be either of the conventional type or of specific subtypes:

### Conventional:

**Figure 2:**
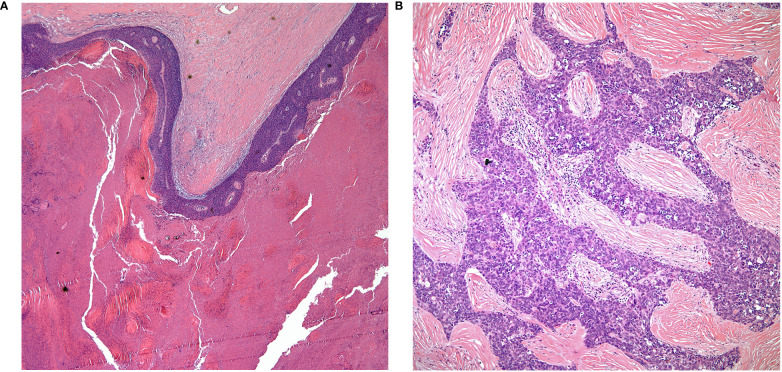
**(A)** keratinizing squamous cell carcinoma, **(B)** thymic carcinoma with loss of organotypical features.

**Figure 3:**
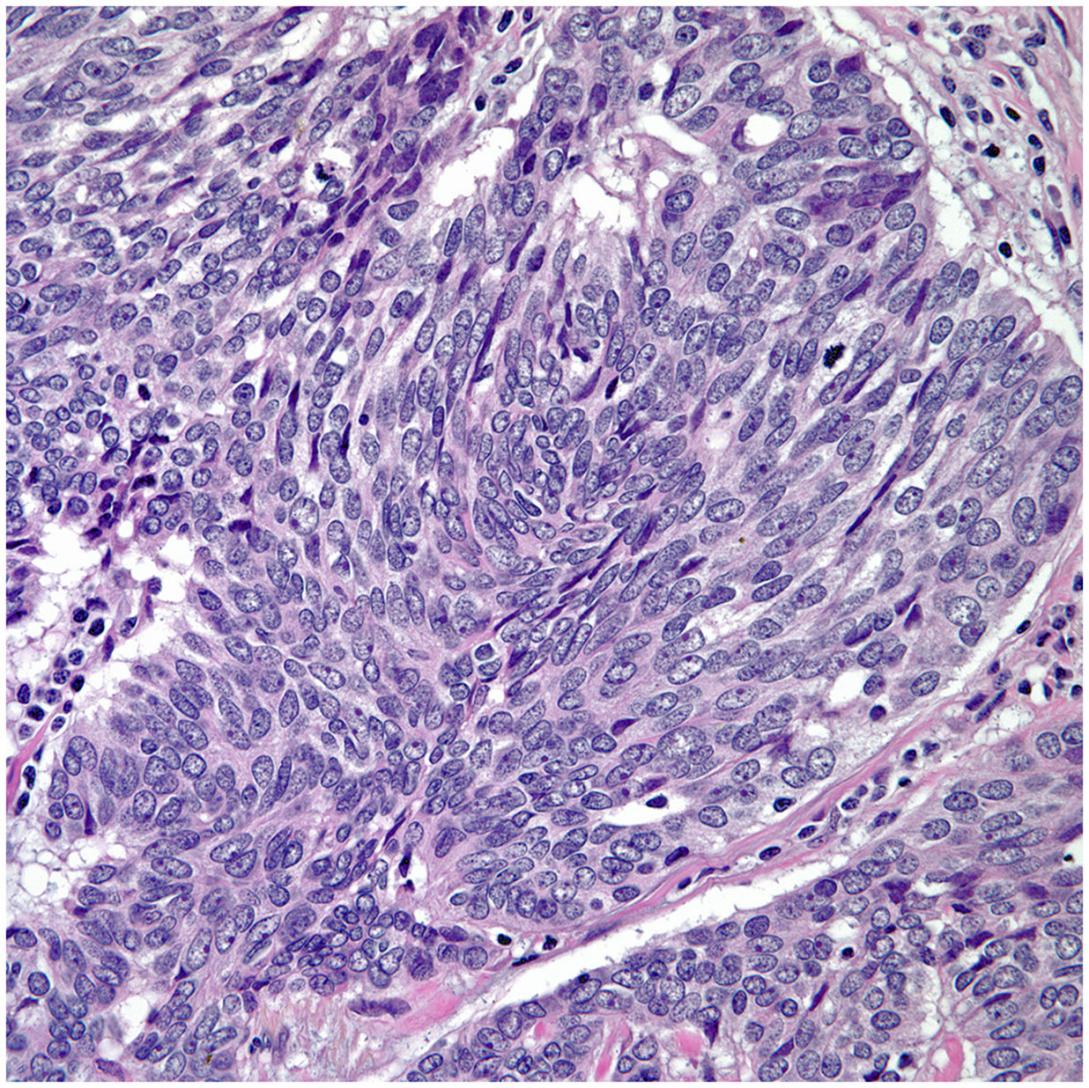
Thymic sarcomaoitd carcinoma.

**Figure 4:**
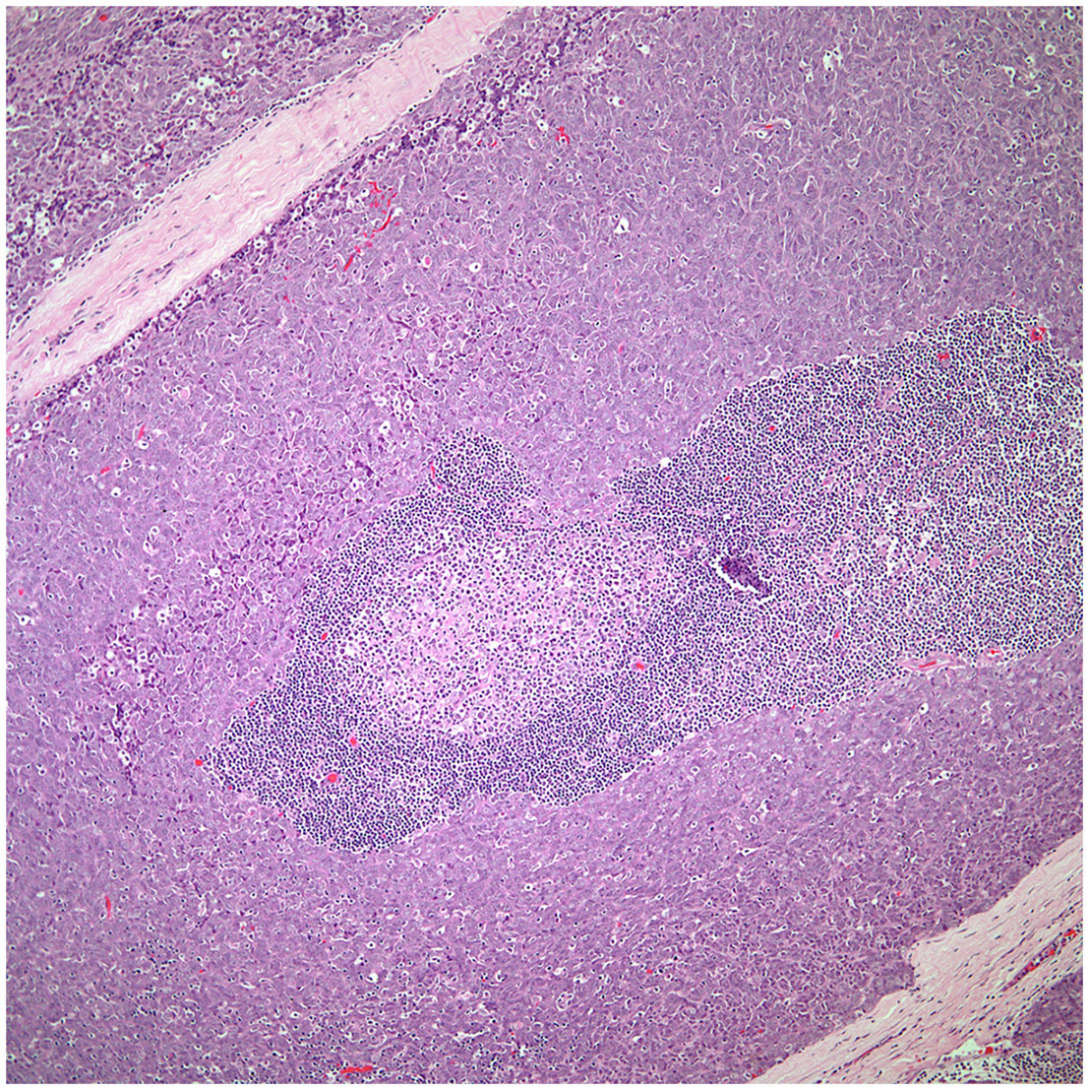
Low power view of a micronodular thymic carcinoma with B-cell lymphoid hyperplasia, note the presence of a germinal center.

• The majority of thymic carcinomas show squamous differentiation and the degree of differentiation varies from well to poorly differentiated tumors (9–13) (**Figures 2A, B**). However, it is also important to keep in mind that all thymomas will also show squamous differentiation because the normal thymic gland also show positive staining in the normal cellularity of the thymus with markers that are commonly used to stain squamous cell carcinomas. Therefore, there is nothing specific for the diagnosis of thymic carcinoma except the presence of an anterior mediastinal mass in the setting of a patient with no other relevant clinical history of malignancy. Over the years, there has been an emphasis on certain histopathological criteria that may be used in the majority of these tumors and that includes the loss of the organotypical features and the presence of overtly cellular atypia and mitotic activity (14, 15).
• Spindle cell morphology is another unusual growth pattern that may be seen in some thymic carcinomas (16) (**Figure 3**). Here the most important consideration would be separating spindle cell thymic carcinoma from spindle cell thymoma or another spindle cell neoplasm of different lineage. However, once again the presence of marked nuclear atypia and mitotic activity will lead to the correct interpretation of thymic carcinoma, while the use of immunohistochemical stains will also be of aid if the consideration is another spindle cell neoplasm. The use of immunohistochemical markers in this particular histology will likely show similar imunophenotype as conventional thymic squamous carcinoma.
• Poorly differentiated (undifferentiated) carcinoma without any morphological or immunohistochemical differentiation (17, 18). This particular group of tumors may show considerable difficulty in diagnosis as there is a need to properly exclude metastatic disease from outside of the mediastinal compartment.
  ○ One important histopathological characteristic of thymic carcinoma is the presence of an inflammatory component in association with the carcinoma, more often composed of plasma cells, which is contrary to thymoma in which the presence of T-cell lymphocytes aids in the diagnosis. However, there are exceptions:
   ▪ Micronodular thymic carcinoma with B-cell lymphoid hyperplasia (**Figure 4**) although rare, represents the counterpart of thymoma with B-cell lymphoid hyperplasia. Both tumors show similar histopathological features and it is the cytological features of the epithelial proliferation, which separates both entities (19, 20).

### Subtypes:

**Figure 5:**
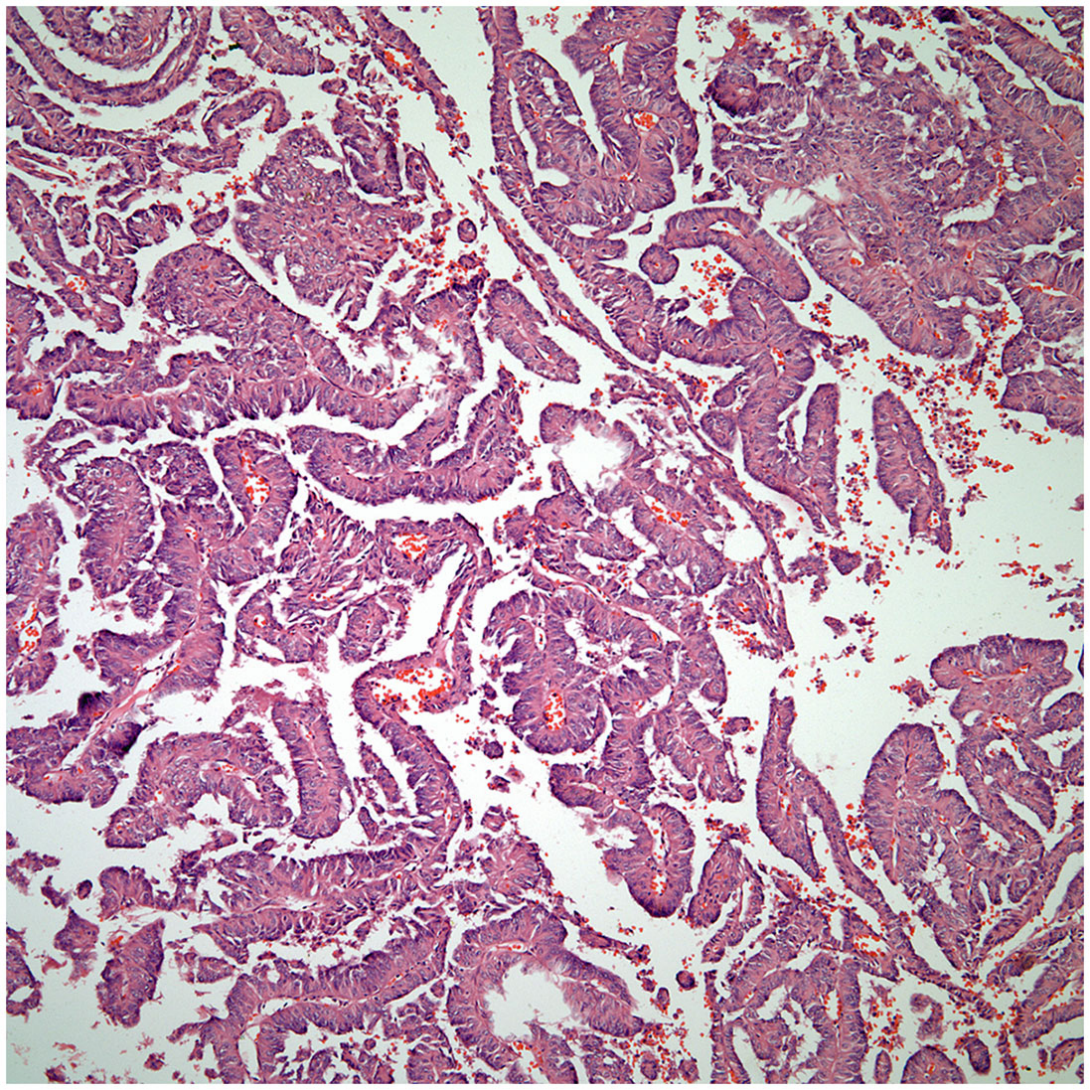
Thymic papillary carcinoma.

**Figure 6:**
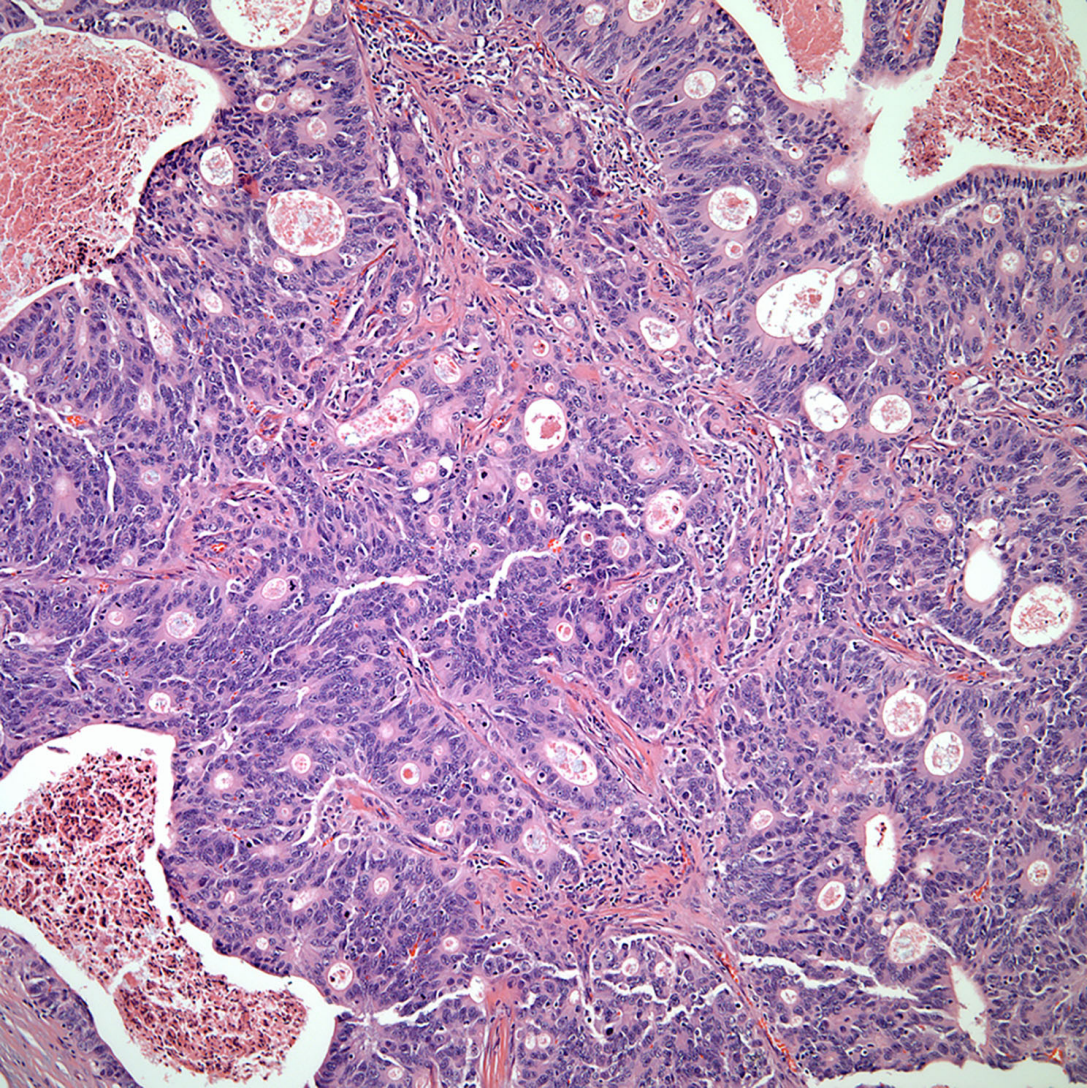
Thymic mucinous adenocarcinoma with “colonic” features.

**Figure 7:**
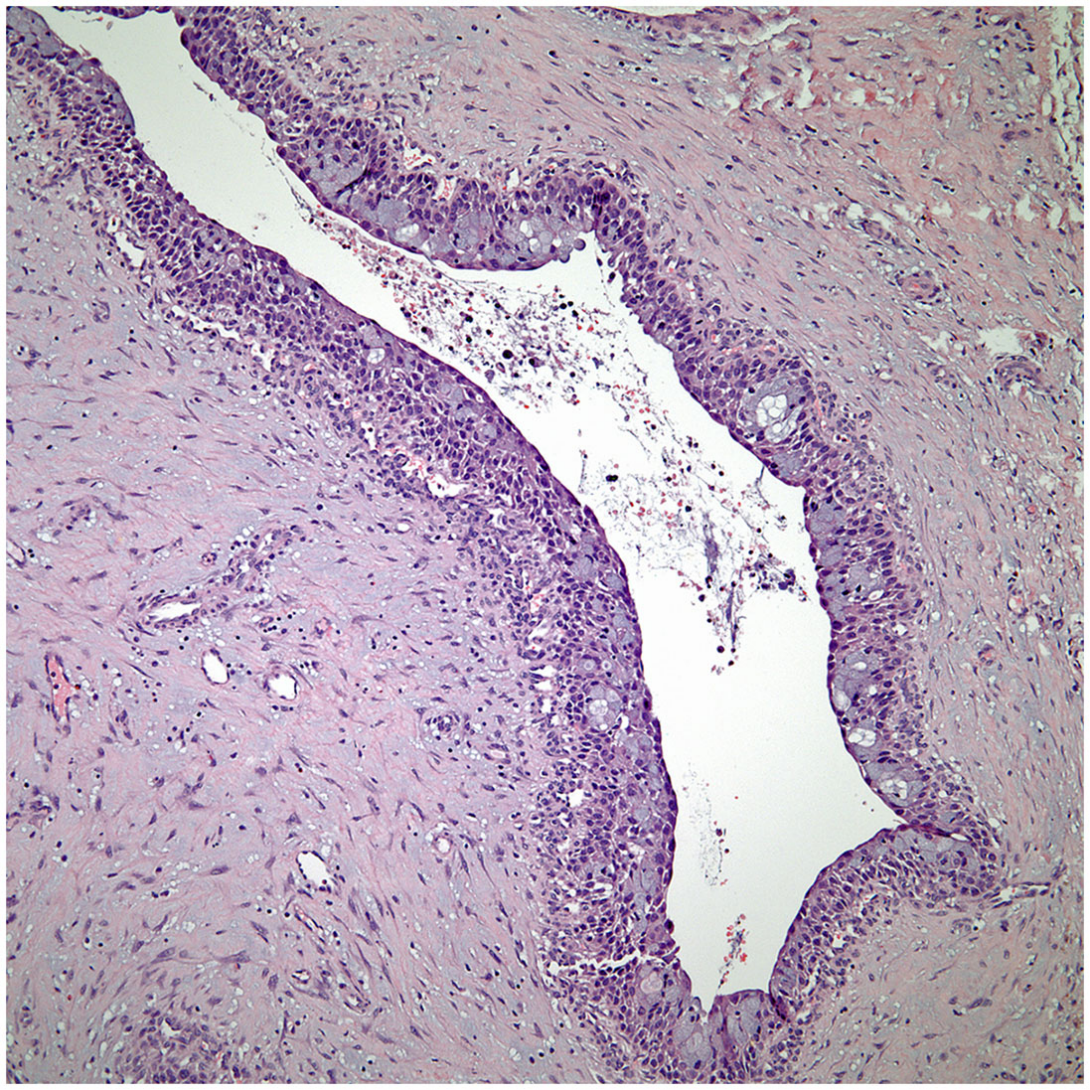
Thymic low-grade mucoepidermoid carcinoma with cystic changes.

**Figure 8:**
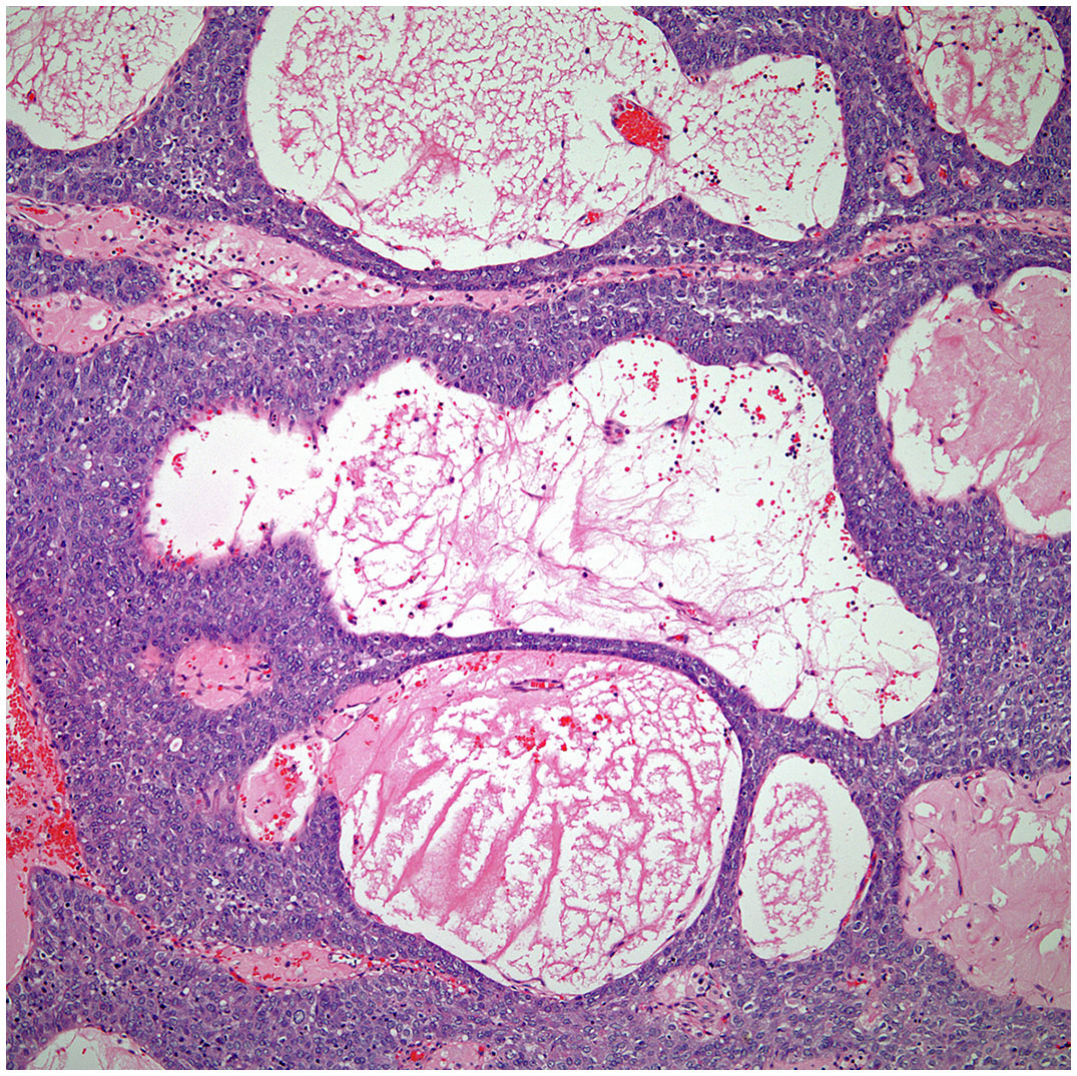
Cystic basaloid carcinoma of the thymus.

▪ The different subtypes that have been described in thymic carcinoma are wide and essentially the tumor may show similar characteristics as other tumors of non-thymic origin. Those growth patterns include:

○ Adenocarcinoma.
  ▪ Even though adenocarcinoma rarely occurs as primary carcinoma of the thymus, the diagnosis of these tumor may pose considerable difficulty as the majority of adenocarcinomas in the thorax are of lung origin. The tumor may show a colonic-like, papillary, and micropapillary growth patterns (**Figures 5**, **6**). In addition, the immunohistochemical profile of thymic adenocarcinoma is also rather non-specific and may be shared with other adenocarcinomas of lung or extra-thoracic origin (21–24).
○ Salivary gland type:
  ▪ Mucoepidermoid carcinoma and to some extent basaloid carcinoma of the thymus show similar features as those described in the salivary gland (**Figures 7**, **8**). Although basaloid carcinoma does not necessarily belong to the group of salivary gland type carcinomas, some of the histopathological features mimic those of the salivary gland. In addition, both tumors may also show cystic changes and both tumors are considered of low-grade malignancy even though mucoepidermoid carcinoma may also be of high-grade histology (25–29). More important is to highlight that the immunohistochemistry of mucoepidermoid carcinoma is also of squamous differentiation and the diagnosis should be based on the presence of mucous producing cells (mucocytes) admixed with the epidermoid proliferation without keratinization. More recent the use of MAML-2 has been correlated with these tumors; however, there are only a few reports of MAML in thymic mucoepidermoid carcinomas to draw more solid conclusions. Needless to say, even though mucoepidermoid and basaloid carcinomas are the most common in this family of tumors, other tumors that have been described include adenoid cystic carcinoma and epithelial myoepithelial carcinoma. However, those two latter tumors are rare in the thymus.

## Immunohistochemical and Molecular Features

One of the bigger issues in understanding the immunohistochemical properties of thymic epithelial neoplasms is the lack of understanding of the immunohistochemical features of the normal thymic gland. Essentially, the normal thymus will show positive staining for markers that are commonly employed in the assessment of squamous carcinomas. Immunostains for p40, keratin 5/6, p63 are positive in the normal thymus (epithelial cells), thymoma, and thymic carcinoma (30). Therefore, those stains have limit value in the setting of thymic epithelial neoplasms. Although CD5 has been considered a good marker for thymic carcinoma, such marker can also show positive staining in atypical thymoma, some conventional thymomas, and other carcinomas of non-thymic origin. On the other hand, some studies on immunohistochemistry have been designed to separate atypical thymomas from thymic carcinoma and although important, as of today, there is not a single immunohistochemical stain that is specific for thymic carcinoma (31–36).

Due to the unusual occurrence of thymic carcinoma, larger studies using molecular techniques is still lacking. However, some authors have investigated the role of EGFR and HER-2, but the results have not been definitive in the role of those biomarkers (37–39).

## Thymic Carcinoma Staging

**Figure 9:**
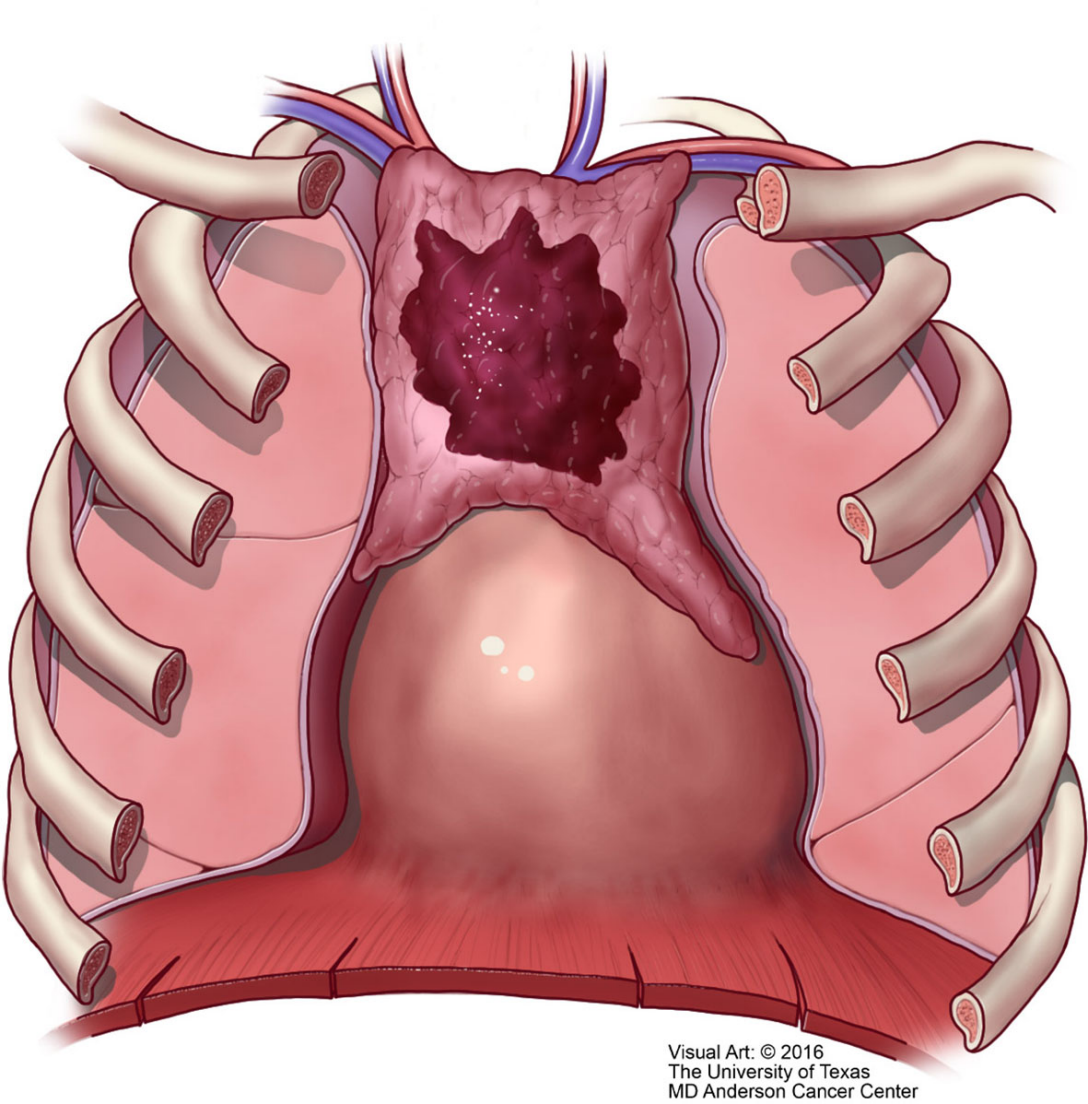
T1 thymic carcinoma in which the tumor is limited to the mediastinal compartment.

**Figure 10:**
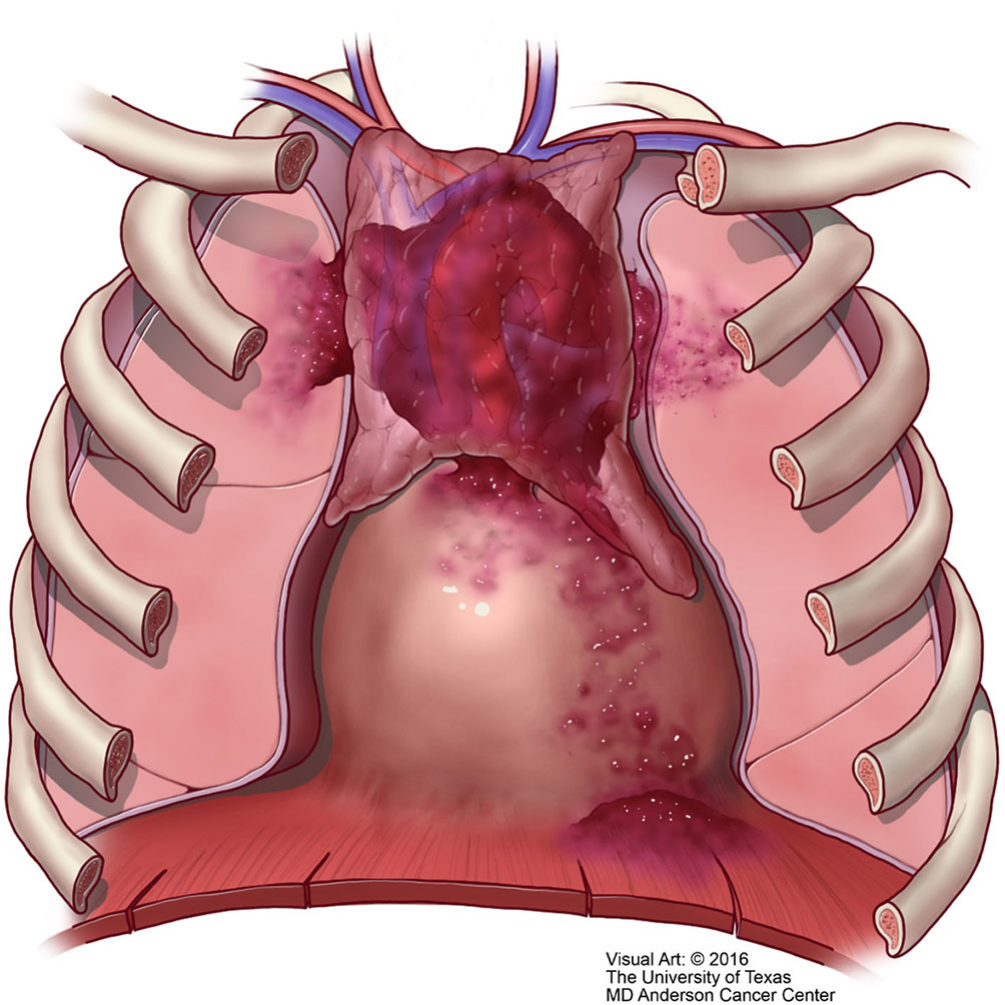
T2 thymic carcinoma in which the tumor invades adjacent structures but within the mediastinal compartment.

**Figure 11:**
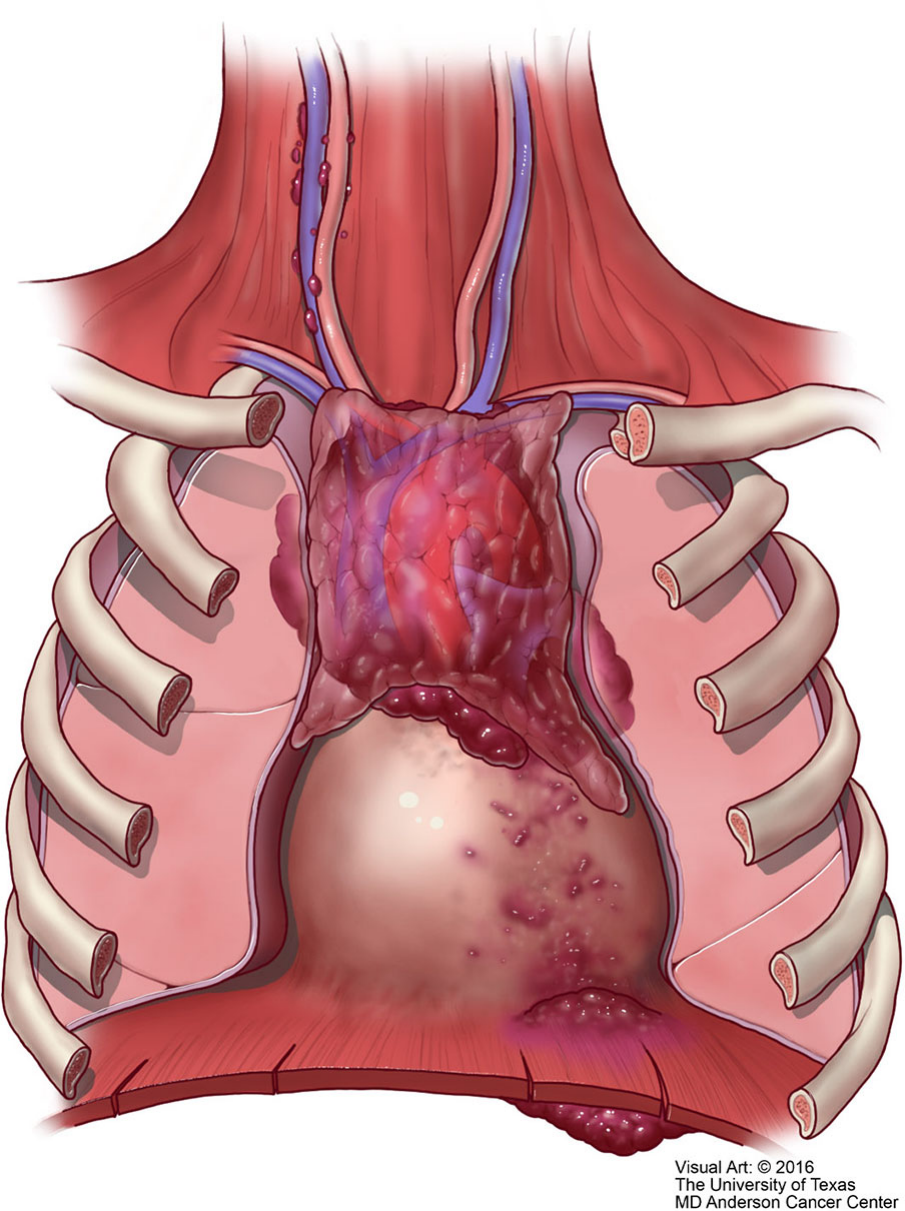
M1 thymic carcinoma in which the tumor invades below the diaphragm or above the thoracic inlet (with permission from Dr. Moran Copyright © 2016).

Unfortunately, over the years the Masaoka staging system that was proposed for thymomas has been the one used for thymic carcinoma (40). However, contrary to the controversy that exists whether the TNM system is applicable to thymoma, we consider that the TNM system is definitely applicable to thymic carcinoma. There is nothing new in that affirmation as there have been several studies using TNM for thymoma that have concluded that TNM is better suited for thymic carcinoma (41–43). However, the authors of the latest version of the WHO book for thoracic tumors (44) have endorsed the use of TNM for all thymic epithelial tumors. We disagree not only with the general use of TNM for all thymic epithelial neoplasm but also to some extent with the definitions in the different stages that have been proposed. Specific reviews on the topic of TNM staging for thymomas have already been presented in the literature (45). In our own experience, we have observed that lymph node metastasis to any lymph node is important in the survival of patients with thymic carcinoma and do not consider that separating lymph nodes between superficial or deep lymph nodes provides a valid statistically meaningful difference in patients with thymic carcinoma. Therefore, based on our own published experienced we have suggested a TNM staging system specifically for thymic carcinoma that is depicted in **Table 1** with the respective illustrative component (**Figures 9**–**11**). Such assessment has been previously illustrated in some publications and textbooks (46–48).

**Table 1:** Suggested TNM staging system for thymic carcinoma.

T1 (Figure 9)	T2 (Figure 10)	T3 (Figure 11)
*Limited to the thymic gland*	*invading visceral pleura* *Lung, pericardium* *Great vessels, chest wall* *Diaphragm.*	*direct extra-thoracic* *tumor extension*
**N0**	**N1**	
*Negative nodes*	*positive thoracic nodes*	
**M0**	**M1**	
*No distant metastasis*	*distant metastasis*	

Groups

Stage I – T1-N0-M0.

Stage II – T2-N0-M0.

Stage III - T3-N0-M0.

Any T, N1, M0.

Any T, any N, M1.

## Conclusions

It is important to highlight that there is nothing specific in the histology of thymic carcinoma. The diagnosis requires careful clinical and radiological assessment. Because of the different growth patterns present in thymic carcinoma, the tumor may also mimic other tumors of non-thymic origin. In addition, in small mediastinoscopic biopsies, the separation between atypical thymoma and thymic carcinoma may not be easily attained. Even though the TNM system is a good approach for the staging of thymic carcinoma, we consider that the one suggested by the WHO for all thymic epithelial neoplasms is not appropriate for all these tumors. Lymph node metastasis in thymic carcinomas regardless of the location of the lymph node is an important characteristic that plays a role in the clinical outcome of these patients.

## Author Contributions

Both of the authors DA and CM contributed in the writing of the manuscript. All authors contributed to the article and approved the submitted version.

## Conflict of Interest

The authors declare that the research was conducted in the absence of any commercial or financial relationships that could be construed as a potential conflict of interest.

## Publisher’s Note

All claims expressed in this article are solely those of the authors and do not necessarily represent those of their affiliated organizations, or those of the publisher, the editors and the reviewers. Any product that may be evaluated in this article, or claim that may be made by its manufacturer, is not guaranteed or endorsed by the publisher.
